# High-Level Production of Amorpha-4,11-Diene, a Precursor of the Antimalarial Agent Artemisinin, in *Escherichia coli*

**DOI:** 10.1371/journal.pone.0004489

**Published:** 2009-02-16

**Authors:** Hiroko Tsuruta, Christopher J. Paddon, Diana Eng, Jacob R. Lenihan, Tizita Horning, Larry C. Anthony, Rika Regentin, Jay D. Keasling, Neil S. Renninger, Jack D. Newman

**Affiliations:** 1 Amyris Biotechnologies, Emeryville, California, United States of America; 2 Departments of Chemical Engineering and of Bioengineering, University of California, Berkeley, California, United States of America; 3 Physical Biosciences Division, Lawrence Berkeley National Laboratory, Berkeley, California, United States of America; 4 Joint BioEnergy Institute, Emeryville, California, United States of America; University of California Los Angeles, United States of America

## Abstract

**Background:**

Artemisinin derivatives are the key active ingredients in Artemisinin combination therapies (ACTs), the most effective therapies available for treatment of malaria. Because the raw material is extracted from plants with long growing seasons, artemisinin is often in short supply, and fermentation would be an attractive alternative production method to supplement the plant source. Previous work showed that high levels of amorpha-4,11-diene, an artemisinin precursor, can be made in *Escherichia coli* using a heterologous mevalonate pathway derived from yeast (*Saccharomyces cerevisiae*), though the reconstructed mevalonate pathway was limited at a particular enzymatic step.

**Methodology/ Principal Findings:**

By combining improvements in the heterologous mevalonate pathway with a superior fermentation process, commercially relevant titers were achieved in fed-batch fermentations. Yeast genes for HMG-CoA synthase and HMG-CoA reductase (the second and third enzymes in the pathway) were replaced with equivalent genes from *Staphylococcus aureus*, more than doubling production. Amorpha-4,11-diene titers were further increased by optimizing nitrogen delivery in the fermentation process. Successful cultivation of the improved strain under carbon and nitrogen restriction consistently yielded 90 g/L dry cell weight and an average titer of 27.4 g/L amorpha-4,11-diene.

**Conclusions/ Significance:**

Production of >25 g/L amorpha-4,11-diene by fermentation followed by chemical conversion to artemisinin may allow for development of a process to provide an alternative source of artemisinin to be incorporated into ACTs.

## Introduction

Malaria causes more than one million deaths annually, with the highest mortality in children younger than five years of age [1]. There are an estimated 350–500 million clinical malaria episodes annually caused by the parasites *Plasmodium falciparum* and *P. vivax*. The most virulent form of malaria is caused by *P. falciparum* which has become resistant to almost all currently used drug therapies [2], [3]. An exception to the pattern of clinical drug resistance seen with older antimalarial drugs is the class of drugs known as ‘artemisinin-based combination therapies’ (ACTs). Artemisinin, a sesquiterpene lactone peroxide with potent antimalarial properties, is extracted from the shrub *Artemisia annua* and combined with one or more other antimalarial drugs to produce ACTs. In 2005 ACTs were recommended for the first-line treatment of uncomplicated falciparum malaria by the World Health Organization (WHO) [3]. Following the WHO recommendation, the price of artemisinin has fluctuated greatly and access to ACTs is still limited in many malaria-endemic countries [4]. Yields of artemisinin from *A. annua* are such that a substantial increase in the amount of plant cultivated would be needed to satisfy the forecasted global demand for artemisinin [5]. New methods for increasing the artemisinin supply would be valuable to stabilize the supply chain and ultimately to increase access to ACTs in developing countries. Chemical synthesis of artemisinin is not practical due to its complexity and low yield [4]. An alternative to total chemical synthesis of artemisinin is the reconstruction of its biosynthetic pathway in microbes leading to the production of precursor molecules that can be converted to artemisinin with relatively few chemical manipulations. Development of a semi-synthetic microbial process for the production of artemisinin would allow for a consistent, second source of the drug to supplement cultivation of *A. annua*.

Historically, heterologous production of small molecules has been hampered by the challenges in expressing complex functional pathways for the production of the non-native molecules. New tools in rapid gene synthesis and metabolite analysis have promoted the field of synthetic biology, which promises advances in pathway re-construction, and strives toward pathway and genome optimization. Recent success in reconstituting heterologous pathways in microorganisms for high-level production of small molecules has demonstrated the feasibility of achieving titers in the g/L range. Examples include production of polyketides such as 6-deoxyerythronolide B [6] and isoprenoids such as amorpha-4,11-diene [7] and lycopene [8] in *Escherichia coli*. Our work focuses on improving heterologous production of the artemisinin precursor amorpha-4,11-diene, which can be converted to artemisinin via chemical transformation.

**Figure 1:**
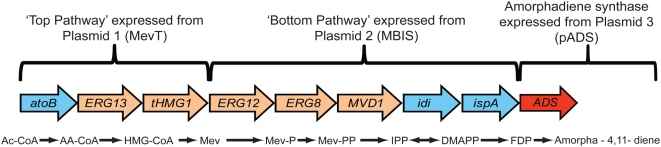
Depiction of the heterologous mevalonate pathway expressed in *E. coli* to produce amorpha-4,11-diene. Genes in blue arrows are derived from *E. coli*, those in brown arrows from yeast, and ADS in red from *A. annua*. pMevT, pMBIS and pADS indicate the arrangement of genes on expression plasmids. Gene names and the enzymes they encode: *atoB*, acetoacetyl-CoA thiolase; *ERG13*, HMG-CoA synthase; *tHMG1*, truncated HMG-CoA reductase; *ERG12*, mevalonate kinase; *ERG8*, phosphomevalonate kinase; *MVD1*, mevalonate pyrophosphate decarboxylase; *idi*, IPP isomerase; *ispA*, farnesyl pyrophosphate synthase. Pathway intermediates: Ac-CoA, acetyl-CoA; AA-CoA, acetoacetyl-CoA; HMG-CoA, hydroxymethylglutaryl-CoA; Mev-P, mevalonate 5-phosphate; Mev-PP, mevalonate pyrophosphate; IPP, isopentenyl pyrophosphate; DMAPP, dimethylallyl pyrophosphate; FDP, farnesyl pyrophosphate; ADS, amorphadiene synthase.

Recently, the expression of a synthetic amorpha-4,11-diene synthase gene along with the mevalonate isoprenoid pathway native to *S. cerevisiae* was engineered in *E. coli* for the production of amorpha-4,11-diene (Figure 1) [9]. Production was increased with the use of a two-phase partitioning bioreactor which captures the product in the organic phase [7]. In this work, we present improvements in amorpha-4,11-diene production in *E. coli* by further strain engineering and fermentation process development. In the original heterologous mevalonate pathway, two key genes (HMG-CoA synthase (HMGS: *ERG13*, Genbank GeneID: 854913) and the catalytic domain of HMG-CoA reductase (tHMGR: *HMG1*, Genbank GeneID: 854900)) were derived from yeast [9]. It was subsequently demonstrated that the activity of tHMGR is insufficient to balance flux in the heterologous pathway leading to a pathway bottleneck [10]. In the current work HMGS and tHMGR were replaced with more active enzymes from *Staphylococcus aureus*, doubling amorpha-4,11-diene production. Amorpha-4,11-diene producing *E. coli* strains were grown in a defined, glucose restricted fed-batch process which achieved cell densities of 90 g/L dry cell weight. Titers were further improved in this process by simultaneously restricting ammonia and carbon. Nitrogen consumption rates depended on the components of the pathway that were expressed, thus the process was modified to accommodate the high production strain containing the modified mevalonate pathway. An amorpha-4,11-diene titer of greater than 25 g/L was achieved in a robust and reproducible fermentation process.

## Results

### Glucose restricted, high cell density bioprocess

**Figure 2:**
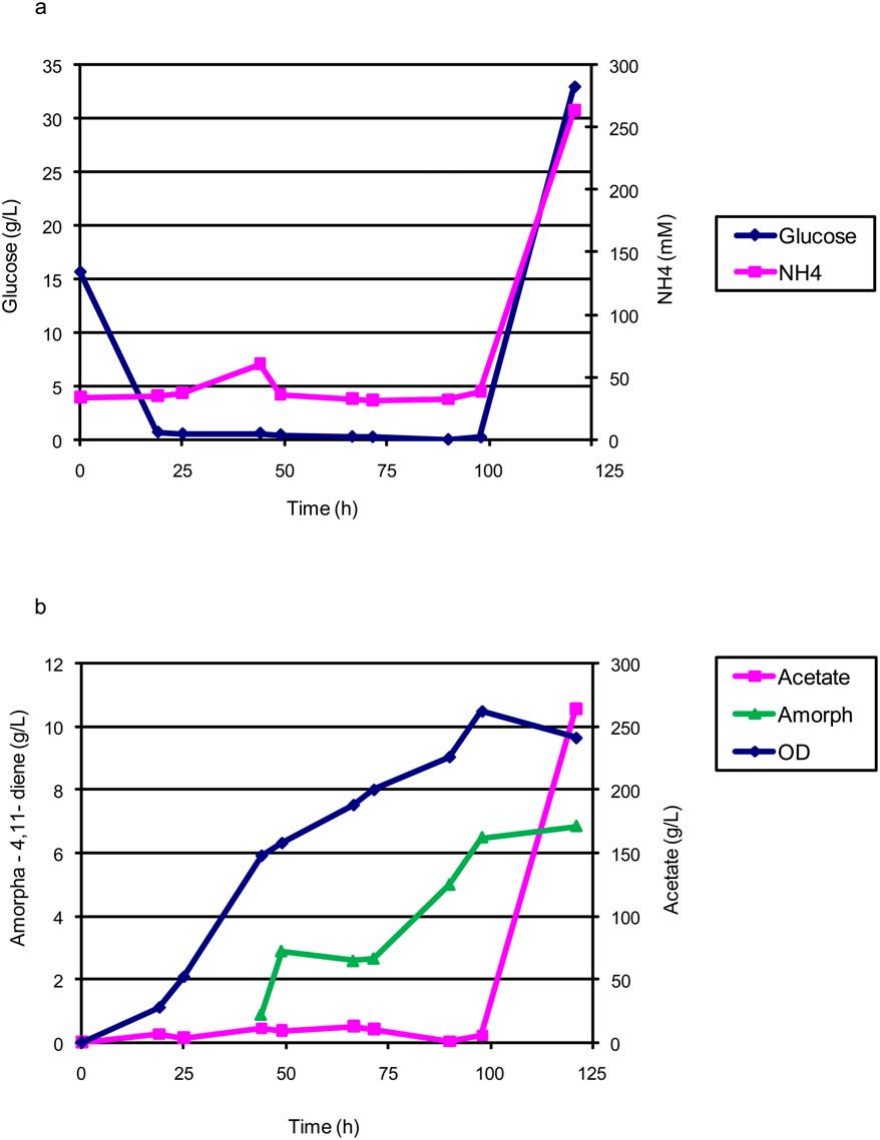
Feed, metabolite, production and cell density for restricted glucose feed (Process A) fed-batch fermentation of *E. coli* strain B32. 2a. Glucose and NH_4_ concentrations. 2b. Cell density, amorpha-4,11-diene production, and acetate concentration.

Newman *et al.* [7] demonstrated production of 0.5 g/L amorpha-4,11-diene in low density fermentations from *E. coli* strain W3110 transformed with plasmids pMevT, pMBIS and pADS [9]. We introduced these plasmids into *E.* coli DH1 [11] to generate strain B32 (Tables 1 and 2). DH1 was chosen as a host strain since the metabolic burden of plasmid presence has been previously characterized [12] and the limited engineering of this strain suggested that it would be a robust host. All subsequently described plasmids were transformed into *E. coli* DH1. To achieve greater volumetric productivity than Newman *et al.* [7], a high-density, glucose-restricted fed-batch fermentation process was developed with ammonia maintained between 30–60 mM until 100 hours (Figure 2a). The sole source of nitrogen for this process was ammonia, which was provided as ammonium hydroxide for pH correction, and ammonium sulfate in the batch and feed media. The initial glucose concentration of 15 g/L was exhausted within 19 hours, at which time an exponential glucose feed with 6 hour doubling time was initiated. Production of amorpha-4,11-diene was initiated with the addition of isopropyl-β-D-1-thiogalactopyranoside (IPTG) at an OD_600_ of ∼30 (20 hours after inoculation). The exponential glucose feed continued until a maximum rate of 31 g/h was achieved, at which point the feed was reduced to 11.7 g/h (3/8 of the maximum) and held constant. Maximum cell density was attained 100 hours after inoculation at an OD_600_ of 260 and the corresponding titer was measured at 6.5 g/L amorpha-4,11-diene (Figure 2b). With this restricted glucose feed process, known as process A, the amount of acetate generated from glycolysis remained close to zero until 100 hours (Figure 2b). Process A, and all processes described below, were run until levels of glucose, acetate, and ammonia were elevated.

**Table 1:** List of Strains.

*Strain*	*Plasmids*
B32	pMevT, pMBIS, pADS
B64	pAM25, pMBIS, pADS
B65	pAM34, pMBIS, pADS
B66	pAM41, pMBIS, pADS
B86	pAM52, pMBIS, pADS

**Table 2:** List of Plasmids.

Plasmid	Description	Reference
pMevT	Expresses the *atoB*, *HMGS (S. cerevisiae ERG13)*, and *tHMGR (S. cerevisiae HMG1)* genes under control of P_LAC_. pACYC184 origin, *Cm^r^*; produces mevalonate from acetyl-coenzyme A	[9]
pAM25	As pMevT, but *atoB*, *HMGS*, and *tHMGR* were codon-optimized for *E. coli* expression and expressed under control of P_LAC(UV5)_	This work
pAM34	As pAM25, but expresses *E. faecalis mvaS* and *mvaE*	This work
pAM41	As pAM25, but *tHMGR* replaced by *S. aureus mvaA*	This work
pAM52	As pAM41, but *HMGS* replaced with *S. aureus mvaS*	This work
pMBIS	Expresses *MK*, *PMK*, *MPD*, *idi*, and *ispA* under control of P_LAC_. pBBR1 replicon, *Tc^r^*; produces farnesyl pyrophosphate from mevalonate.	[9]
pADS	Expresses *A. annua* amorphadiene synthase under control of P_LAC_	[9]

### Glucose and ammonia restriction at high cell density

**Figure 3:**
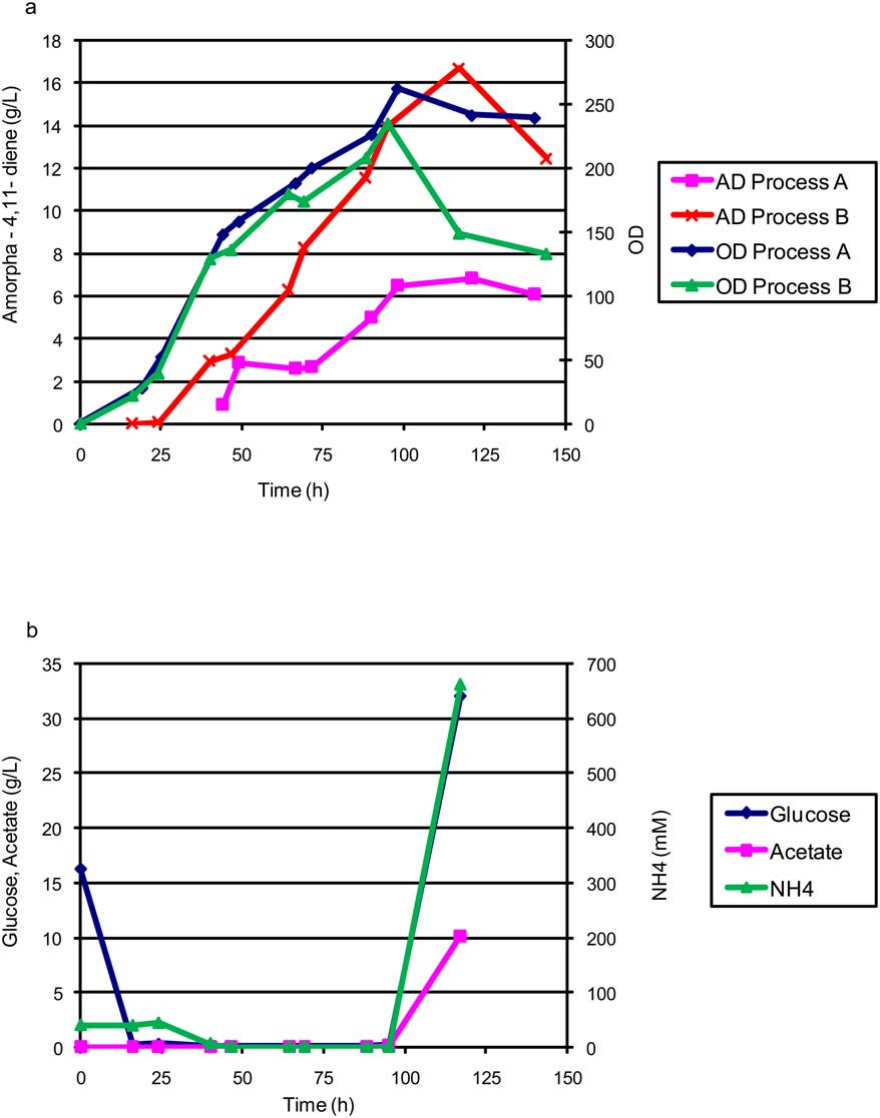
Feed, metabolite, production and cell density for restricted glucose and nitrogen feed (Process B) fed-batch fermentation of *E. coli* strain B32. 3a. Comparison of cell density and amorpha-4,11-diene production in process A (glucose restricted) and process B (glucose and nitrogen restricted) fermentations. 3b. Glucose, acetate and ammonia concentrations.

To test the effect of nitrogen restriction on cell growth and production of amorpha-4,11-diene, process A was modified whereby the ammonium sulfate was left out of the feed (process B). The ammonia in the medium was allowed to decrease to zero though nitrogen flow into the bioreactor was still positive due to base addition. The carbon consumption in process B was similar to that of process A (data not shown). Once the initial glucose was depleted, the glucose concentration was maintained at zero, thus preventing any accumulation of acetate in the culture (Figure 3b). Ammonium sulfate in the batch medium was utilized in the first 40 hours and the amount of ammonium hydroxide added to correct the pH thereafter did not result in ammonia accumulation until 95 hours (Figure 3b) while supporting maximum cell density of 235 OD_600_ (Figure 3a). Similar cell growth between processes A and B confirmed sufficient supply of nitrogen from the base to build cell mass in process B despite the fact that the measured ammonia in the culture was zero. The difference in amorpha-4,11-diene production between the two runs was evident after 50 hours where the production profiles diverged (Figure 3a). This correlated well with the ammonia concentrations measured in the culture between 50 hours and 100 hours. In process A, ammonia was in excess at 40 mM whereas in process B the ammonia concentration was negligible during most of the production period. Process B with strain B32 achieved a peak amorpha-4,11-diene titer of 16.7 g/L at 120 hours, a 2.5-fold improvement in titer compared to process A.

### Improvements in amorpha-4,11-diene production through strain engineering

**Figure 4:**
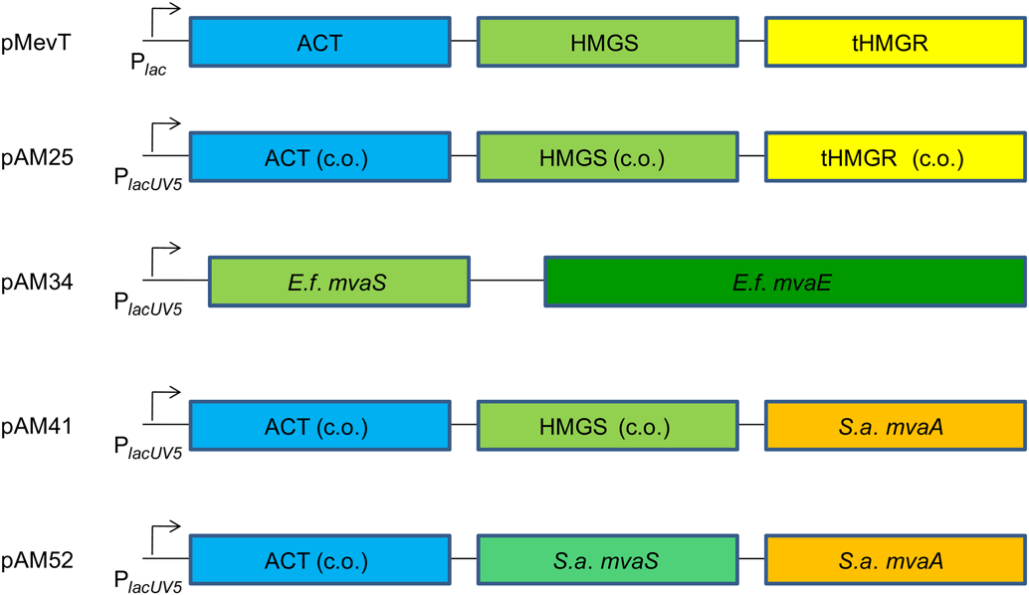
Schematic of operons in plasmids encoding the first 3 enzymatic activities of the synthetic mevalonate pathway. ACT, *E. coli* acetoacetyl-CoA thiolase (*atoB*); HMGS, *S. cerevisiae* HMG-CoA synthase (*ERG13*); tHMGR, truncated *S. cerevisiae* HMG-CoA reductase (*HMG1*); *E.f. mvaS*, *E. faecalis* HMGS; *E.f. mvaE*, *E. faecalis* acetoacetyl-CoA thiolase / HMGR; *S.a. mvaS*, *S. aureus* HMG-CoA synthase; *S.a. mvaA*, *S. aureus* HMG-CoA reductase; (c.o), codon-optimized for *E. coli* expression.

**Figure 5:**
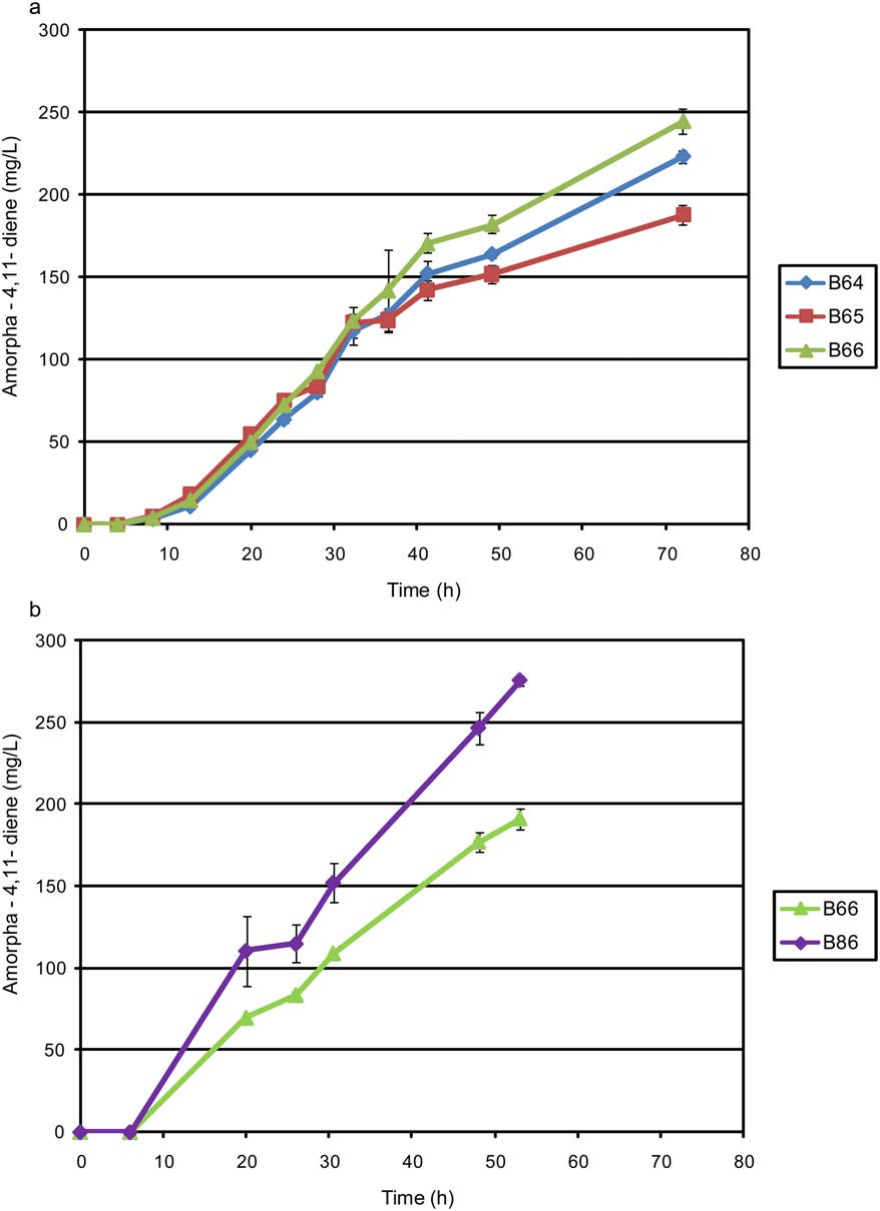
Production of amorpha-4,11-diene in shake-flask cultures by strains expressing different HMGR and HMGS enzymes. 5a: Production of amorpha-4,11-diene by shake-flask cultures of strains B64, B65 and B66. B64 (pAM25, codon-optimized pMevT: blue curve), B65 (pAM34, *E. faecalis mvaE mvaS*: red curve), B66 (pAM41, *S. aureus mvaA*: green curve). 5b: Production of amorpha-4,11-diene by shake-flask cultures of strains B66 and B86. B66 (pAM41, *S. aureus mvaA* green curve), B86 (pAM52, *S. aureus mvaS mvaA*: purple curve).

Strain B32 (Table 1) contains a heterologous mevalonate pathway consisting of genes derived from yeast, including a truncated version of HMGR (tHMGR) expressing solely the C-terminal catalytic domain, and HMGS (Figure 4, pMevT. Table 2). Pitera *et al.* [10] showed that accumulation of the pathway intermediate HMG-CoA limited flux through the pathway and could result in growth inhibition due to the apparent toxicity of this intermediate. An initial attempt to improve flux through the mevalonate pathway was made by re-synthesizing the MevT operon, which expresses the first three genes of the mevalonate pathway, with genes codon-optimized for expression in *E. coli*. The wild-type lac promoter was also replaced with the two-fold stronger lacUV5 promoter [13], which also displays significantly lower sensitivity to intracellular levels of cAMP, thereby being less sensitive to glucose repression. The plasmid bearing the codon optimized version of the MevT operon, pAM25, is shown schematically in Figure 4 along with all top pathway plasmids described below. Production of amorpha-4,11-diene from strain B64 containing pAM25, pMBIS and pADS was 1.5× higher than that from DH1 strain B32 [14]. Moreover, since the design of the operon enabled more convenient DNA manipulation, pAM25 was used as a basis for all subsequent pathway engineering. To further alleviate the HMGR pathway bottleneck more efficient HMGR enzymes were sought to prevent the accumulation of HMG-CoA and increase flux through this step in the pathway. Two HMGR enzymes from gram-positive bacteria were investigated by replacement of yeast tHMGR expressed in pAM25. The chosen gram-positive enzymes were HMGR from *S. aureus* encoded by *mvaA* [15] (Genbank GeneID: 2861328), and the fused acetoacetyl-CoA thiolase /HMGR from *Enterococcus faecalis* encoded by *mvaE* [16] (Genbank GeneID: 1200264). In the case of the reconstructed operon incorporating *mvaE*, the entire codon-optimized MevT operon was replaced with *E. faecalis* genes *mvaS* (encoding HMGS [17]; Genbank AF290092) and *mvaE* (pAM34; Figure 4). The production of amorpha-4,11-diene by strains B64 (top pathway encoded by pAM25 expressing acetoacetyl-CoA thiolase, HMGS and HMGR from the codon-optimized MevT operon [9]), B65 (top pathway encoded by pAM34 expressing *E. faecalis mvaE mvaS*), and B66 (top pathway encoded by pAM41 expressing acetoacetyl-CoA thiolase, HMGS, and *S. aureus mvaA*), (Figure 4), pMBIS and pADS, was compared during growth in shake flasks for 72 hours (Figure 5a). The highest concentration of amorpha-4,11-diene (244 mg/L after 72 hours growth) was produced by strain B66 expressing the mevalonate pathway incorporating *S. aureus mvaA*, followed by B64 expressing the codon-optimized yeast-derived [9] mevalonate pathway (223 mg/L amorpha-4,11-diene). Strain B65 expressing *E. faecalis mvaE mvaS* produced only 188 mg/L amorpha-4,11-diene. The mevalonate pathway incorporating the *S. aureus* HMGR encoded by *mvaA* (strain B66) was selected for further study. In view of the superiority of the *S. aureus* HMGR for the production of amorpha-4,11-diene in *E. coli* we wished to test the effect of substituting the yeast HMGS with its *S. aureus* equivalent encoded by *mvaS*. This was accomplished by site-directed mutagenesis of pAM41 to substitute the yeast HMGS with *S. aureus mvaS*, generating pAM52 (Figure 4). Amorpha-4,11-diene production in shake flasks from the resultant strain (B86, containing pAM52, pMBIS and pADS) was compared with its progenitor strain B66 (containing pAM41, pMBIS and pADS). Amorpha-4,11-diene production (Figure 5b) was higher in B86 than B66, demonstrating the superiority of the *S.aureus mvaS*+*mvaA* combination compared to the yeast HMGS+*S. aureus mvaA* combination.

### Development of a fermentation process to produce 25 g/L amorpha-4,11-diene

**Figure 6:**
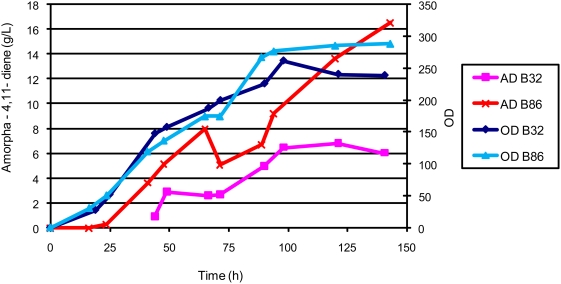
Cell growth and amorpha-4,11-diene production for strains B32 and B86 in process A.

**Figure 7:**
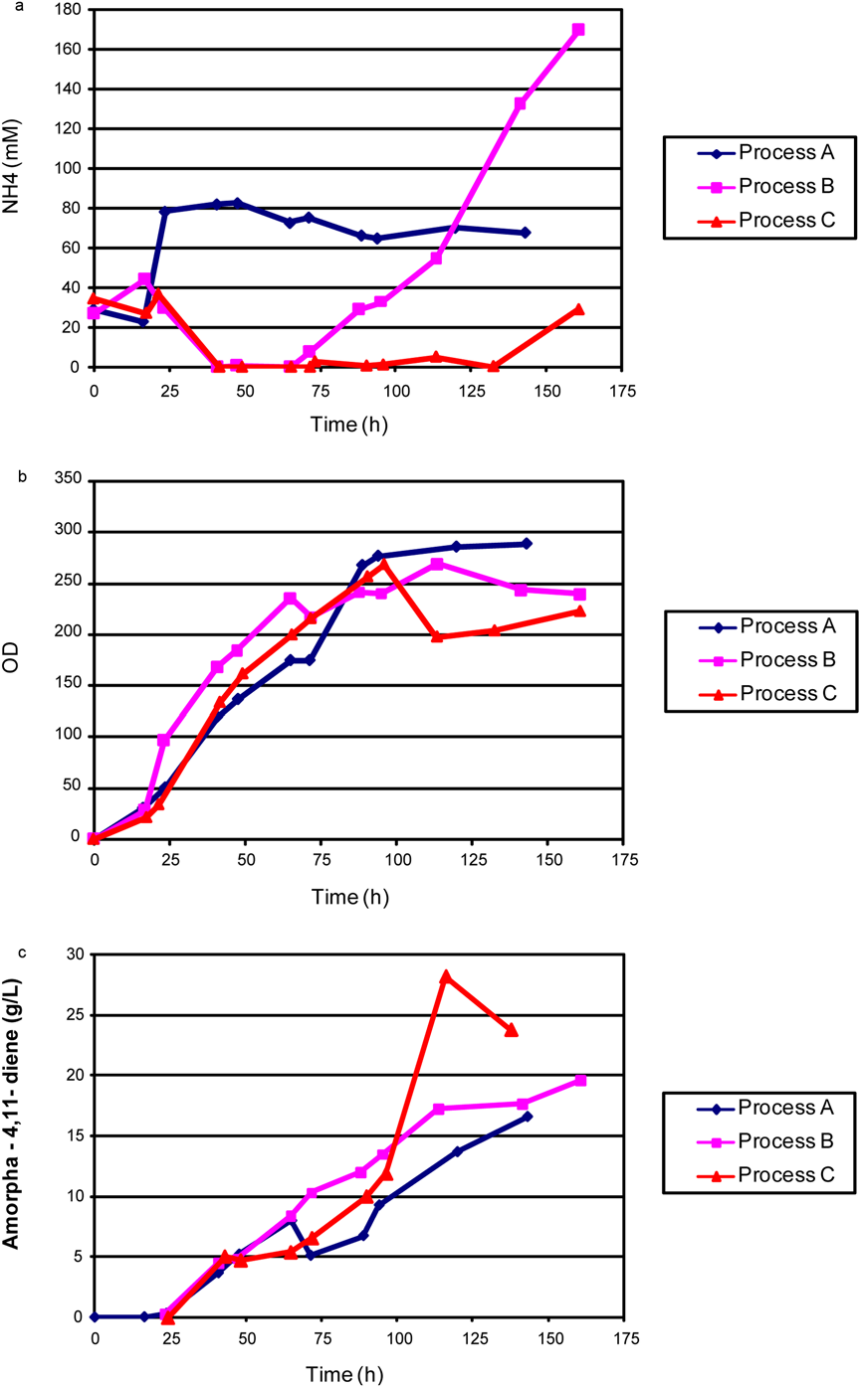
Comparison of ammonia concentration, cell growth and amorpha-4,11-diene production in fed-batch processes A (restricted glucose), B (restricted glucose and nitrogen) and C (restricted glucose and nitrogen with NaOH pH control) for strain B86. 7a: Ammonium concentration. 7b: Cell growth. 7c: Production of amorpha-4,11-diene.

Production of amorpha-4,11-diene was compared between strains B32 and B86 in bioreactors using process A. While growth of the two strains was comparable, strain B86 produced 2.5-times more amorpha-4,11-diene than B32, reaching 16.5 g/L at 150 hours (Figure 6). The ammonia concentration remained steady between 60–80 mM in the B86 culture in this fermentation process (Figure 7A)

To determine whether ammonia restriction is a generally applicable approach in improving the production of amorpha-4,11-diene in *E. coli*, strain B86 was tested in process B. With no ammonium sulfate in the feed, the ammonia concentration was undetectable at 42 hours, but only remained restricted for 24 hours (Figure 7a). The subsequent accumulation of ammonia up to 170 mM was due to ammonium hydroxide addition for pH maintenance and also the slower ammonia consumption rate of the strain. Strain B86 in process B produced 16.5 g/L amorpha-4,11-diene at 140 hours, which is comparable to the production of B86 in process A (Figure 7c) and B32 in process B. No substantial improvement in amorpha-4, 11-diene production was observed when ammonia was restricted for only 24 hours during the production phase.

In view of the superior performance of strain B86 in flask production experiments (Figure 5) and fermentation process A, and the known limitations of the yeast-derived tHMGR [10], [18], strain B86 was chosen for further development. To extend the period of nitrogen restriction with strain B86, the ammonia-restricted process (process B) was modified so that ammonium hydroxide in the base solution was reduced from 9.9 M to 4.9 M with the addition of 1.3 M NaOH (process C). By exchanging the concentrated ammonium hydroxide base solution at 72 h with the dilute solution, constant nitrogen restriction was attained through most of the production period (Figure 7a). The three processes attained similar cell densities, although the density in process C was somewhat reduced after 100 h (Figure 7b). Nonetheless, the production of 29.7 g/L of amorpha-4,11-diene was achieved with the successful restriction of ammonia in process C (Figure 7c). Further reduction of ammonia delivery to the bioreactors by increasing the use of NaOH for pH correction did not improve production. Reducing the ammonia delivery below the rate that just maintained an undetectable concentration led to accumulation of acetate and lower titers (data not shown).

**Figure 8:**
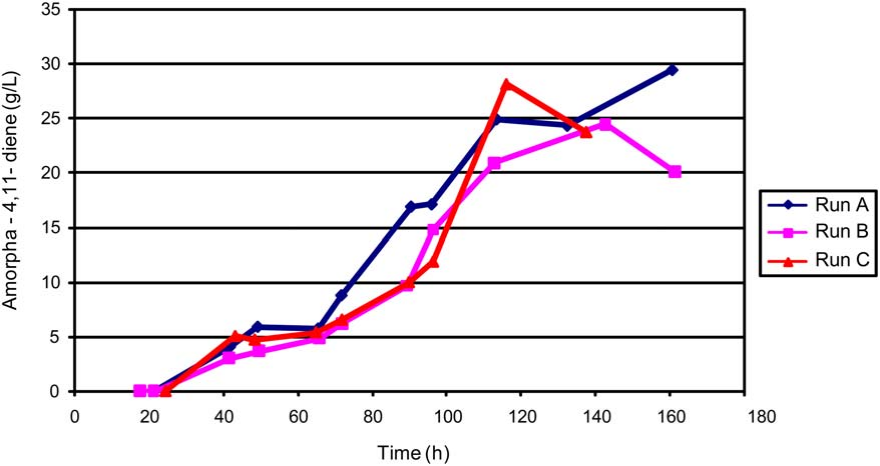
Amorpha-4,11-diene production in triplicate fed-batch process C fermentations with strain B86.

The process was replicated three times to determine the production variability of strain B86 in process C. The three runs displayed similar cell growth, consistently yielding cell densities over 220 OD_600_ (equivalent to 88 g/L dry cell weight). An average titer of 27.4±2.1 g/L amorpha-4,11-diene was attained from these runs (Figure 8) which is a 50-fold improvement over the previously reported highest titer [7].

## Discussion

Production of an artemisinin precursor by fermentation followed by an inexpensive chemical conversion to an active anti-malarial compound is an attractive supplement to current drug production methods. Fermentation is a controlled process, amenable to scale-up, and a more reliable production method than agricultural sources, which typically have planting cycles in excess of 12–18 months [5]. The high titers of amorpha-4,11-diene reported here are a promising step towards development of a reliable second source for the economical production of artemisinin.

Prior fermentation development on amorpha-4,11-diene producing *E. coli* utilized an undefined excess carbon bioprocess [7]. We transformed the plasmids encoding the heterologous farnesyl pyrophosphate-production pathway used previously [7], [9] into a robust strain of *E. coli* and showed a ten-fold increase in production in a carbon-restricted, chemically defined process compared to [7] (Figure 2b). Maintaining an ammonia concentration at or near zero throughout much of the fermentation further improved titers (Figure 3b).

Strain improvement was an integral component of enhancing the production of amorpha-4,11-diene. Pitera *et al.* [10] showed that buildup of HMG-CoA could be detrimental to flux through the heterologous mevalonate pathway and can inhibit cell growth in some situations. Kizer *et al.* [18] subsequently demonstrated that buildup of HMG-CoA inhibits fatty acid biosynthesis, leading to generalized membrane stress. It seemed likely that the yeast-derived tHMGR, which is a truncated enzyme containing only the C-terminal catalytic domain, was limiting flux through the heterologous mevalonate pathway. We hence sought alternative HMGRs that would overcome the flux limitation imposed by tHMGR. Tabata and Hashimoto [19] described a process for the production of mevalonate using *E. coli* expressing a heterologous mevalonate pathway derived from *E. faecalis* that achieved production of 47 g/L mevalonate. The *E. faecalis* HMGR encoded by *mvaE* is part of a fused enzyme, having both acetoacetyl-CoA thiolase and HMGR activities. The *E. faecalis* HMGR encoded by *mvaE* has the enzymatic properties of a class II HMGR [16] but has significantly lower V_max_ than other class II HMGR enzymes [20]. In view of the lower V_max_ of *E. faecalis* HMGR encoded by *mvaE*, we measured production of amorpha-4,11-diene in shake flask cultures following replacement of tHMGR with either *mvaE* or *S. aureus* HMGR encoded by *mvaA* [15]. We found rank order production of amorpha-4,11-diene to be *mvaA*>tHMGR>*mvaE* (Figure 5a). This result was surprising as HMGR enzyme assays showed cell lysates from strain B65 expressing *mvaE* to contain several-fold higher specific activity than lysates from strain B64 expressing tHMGR (data not shown). It may be that the acetoacetyl-CoA thiolase activity of *mvaE* or the HMGS encoded by *E. faecalis mvaS* limits flux through the mevalonate pathway, though the production of 47 g/L mevalonate by a strain expressing *mvaE* and *mvaS* [19] suggests that this is not the case.

In an effort to further improve production of amorpha-4,11-diene from strain B66 (expressing *S. aureus mvaA*) we replaced the yeast HMGS with *S. aureus mvaS* [21] resulting in a >40% increased production in flasks from strain B86. Thus, replacement of the yeast enzymes HMGS and HMGR with their equivalent enzymes from *S. aureus* resulted in a significant increase in amorpha-4,11-diene production from the heterologous mevalonate pathway.

The titers were improved further by optimizing nitrogen delivery in the fermentation process, so that the ammonia concentration in the medium was maintained at undetectable concentrations through most of the process. In microorganisms that naturally produce secondary metabolites, such as actinomycetes, high concentrations of ammonia frequently down-regulate production [22]. Down-regulation of secondary metabolite production in a heterologous host by ammonia [6], [23] might be expected in cases where the genes for the heterologous pathway come from a closely related organism using similar regulatory networks sensitive to ammonia. Production of epothilones from a *Sorangium cellulosum* pathway transferred into the heterologous host *Myxococcus xanthus* was very sensitive to ammonia in the medium, though the native producer was more sensitive [23]. Similarly, production of 6-deoxyerythronolide B was reduced in the heterologous host *E. coli* by ammonia concentrations as low as 25 mM in shake flasks [6]. The sensitivity of 6-deoxyerythronolide B production in *E. coli* to ammonia is difficult to explain because gene expression in that system is induced with IPTG rather than a native promoter [6]. Growth of *E. coli* is not inhibited until ammonia concentrations reach levels >170 mM [24], ruling out simple growth inhibition as the mechanism of ammonia sensitivity.

An explanation for the increase in production in the ammonia restricted process is likely related to carbon flow. Glucose restricted feeds effectively control the production of acetate in high density fermentations by limiting the rate of cell growth [25]. However, since the rate of amorpha-4,11-diene biosynthesis in *E. coli* depends on the glucose feed to supply the metabolic precursor acetyl-CoA, product biosynthesis rates are influenced by feed rates. The nitrogen restriction could reduce amino acid and protein biosynthesis in the cells and increase carbon availability for amorpha-4,11-diene production.

Simultaneous restriction of two major nutrients in a fermentation process poses some challenges. In the event that one component is restricted too tightly, the other might accrue in the fermentation broth. The process here where both nitrogen and carbon were maintained at or near zero throughout much of the fermentation process was achieved by taking advantage of nitrogen supply via pH control. Because much of the nitrogen used in the process was supplied by the automatic addition of base for pH control, it was possible to reduce nitrogen flow into the bioreactor by diluting the ammonium hydroxide in the base feed with sodium hydroxide. Another approach for ammonia control is to use an ammonia gas sensing electrode. This method successfully maintained ammonia between 10–100 mM in a *S. cerevisiae* process for production of ergosterol, improving production 1.3-fold [26]. For processes where nitrogen is supplied in another form, careful consumption measurements would be required to implement a process with double restriction, and the same holds true for simultaneous restriction of glucose and other nutrients such as phosphate or iron.

The development of a strain of *E. coli* and an accompanying process capable of producing over 25 g/L amorpha-4,11-diene in fed-batch fermentation is a significant step towards the development of a commercial process for the semi-synthetic production of artemisinin. Full commercialization of the described process would require removal of antibiotic selection, for instance by integration of the heterologous mevalonate pathway into the genome [27], and use of an alternative induction system that did not require the expensive inducer IPTG. The amorpha-4,11-diene produced by fermentation could potentially be chemically oxidized to artemisinic acid, which in turn can be converted to artemisinin [28]. Alternatively, oxidation of amorpha-4,11-diene to artemisinic acid could be accomplished *in vivo* by expression of *CYP71AV1*, the cytochrome P450 from *A. annua* [29], [30] in an amorpha-4,11-diene-producing *E.coli* strain. Chang *et al.* [31] demonstrated that *CYP71AV1* can be functionally expressed in *E. coli* with its cognate P450 reductase, though only low amounts of artemisinic alcohol were produced. Modifications to the N-terminal transmembrane sequence of *CYP71AV1*, along with changes in expression vector, host strain, and culture conditions, resulted in production of 105 mg/L of artemisinic acid. Subsequent development of amorpha-4,11-diene producing *E. coli* expressing *CYP71AV1* and its cognate reductase has allowed a significant improvement in production of artemisinic acid (D. Pitera, Pers. Comm.). An alternative method of microbial production of amorpha-4,11-diene or artemisinic acid is the use of engineered yeast (*Saccharomyces cerevisiae*). Ro *et al.* [29] produced 153 mg/L of amorpha-4,11-diene in yeast cultures by expression of ADS and manipulation of the native mevalonate pathway. Expression of *CYP71AV1* in this yeast strain resulted in production of 115 mg/L artemisinic acid. The strain described by Ro *et al.* [29] was subsequently shown to be capable of producing 2.5 g/L artemisinic acid in a novel fermentation process [32]. The work described here, enabling the production of over 25 g/L amorpha-4,11-diene from *E. coli* combined with chemical oxidation and transformation to artemisinin is one option for the semi-synthetic production of artemisinin, which will need to be compared for feasibility of commercial production with a yeast process capable of producing lower concentrations (2.5 g/L) of artemisinic acid. A second source of artemisinin to stabilize the price and increase the supply of ACTs for the treatment of malaria in the developing world is highly desirable, and this work describes progress towards an option for the provision of such an alternative supply.

## Methods

### Strains

*E. coli* DH1 was used as the isoprenoid expression strain. The B32 strain contained plasmids encoding the heterologous mevalonate pathway previously described by Martin *et al.* [9]. Construction of the *E. coli* codon-optimized MevT operon is described by Anthony *et al.* [14]. Codon-optimized MevT (referred to as MevT66 by Anthony *et al.* [14]) was ligated into *EcoRI*+*HindIII* digested pAM29 [14] to generate pAM25. For construction of pAM34, *Enterococcus faecalis mvaS* was amplified from genomic DNA of *E. faecalis* ATCC 700802 using primers TATAGAATCTTAAGGAGGATATTTAGATGACAATTGGGATTGATAAAATTAG and TTTGGATCCTTAGTTTCGATAAGAGCGAACGG with Phusion™ DNA polymerase (New England Biolabs; all amplifications used this enzyme according to manufacturer's instructions) and the following conditions: 1 cycle 98°C, 30 s; 30 cycles 98°C, 30 s/ 55°C, 20 s/ 72°C, 90 s; 1 cycle 72°C, 10 m. *E. faecalis mvaE* was similarly amplified with primers TATGGATCCTAAGGAGGATATTTAGATGAAAACAGTAGTTATTATTGATGC and AGCTAAGCTTTTATTGTTTTCTTAAATCATTTAAAATAGC. Amplicons were ligated into *SmaI* digested pBluescript II KS+ (Stratagene) and the DNA sequence verified. *E. faecalis mvaS* and *mvaE* were excised from pBluescript II KS+ with *EcoRI*+*BamHI* and *BamHI*+*HindIII* respectively and the excised amplicons were ligated into *EcoRI*+*HindIII* digested pAM29 [14] to generate pAM34. HMGR from pAM25 was replaced with *Staphylococcus aureus mvaA* (HMGR) to generate pAM41. *S. aureus mvaA* was amplified from genomic DNA of *Staphylococcus aureus* subsp. *aureus* ATCC 700699D with the primers GCTACTAGTAGGAGGAAAACATCATGCAAAGTTTAGATAAGAATTTCCG and GCTTCTAGACTATTGTTGTCTAATTTCTTGTAAAATGCG using the same reaction conditions used to amplify *E. faecalis mvaE* and *mvaS*. The amplified *mvaA* fragment was digested with *SpeI* and ligated into the *SpeI+HincII* digested pAM25 so as to create pAM41 with the operon *atoB-ERG13-mvaA*. *mvaS* was similarly amplified with the primers GAACTGAAGATCTAGGAGGAAAGCAAAATGACAATAGGTATCGACAAAATAAACT and TTGCATGATGTTTTCCTCCTACTAGTTACTCTGGTCTGTGATATTCGCGAAC. *ERG13* in the *atoB-ERG13-mvaA* operon of pAM41 was replaced with *mvaS* by the method of Geiser *et al.* [33] to generate the *atoB-mvaS-mvaA* operon in pAM52.

### Flask growth conditions and seed cultures

Seed cultures for fed-batch cultivation were prepared by inoculating 1 mL of frozen cells (20% (v/v) glycerol) of *E. coli* strain B32 or B86 into a flask containing 50 mL of M9 medium. M9 medium contained (per L) 8 g glucose, 12.8 g Na_2_HPO_4_·7H_2_O, 3 g KH_2_PO_4_, 0.5 g NaCl, 1 g NH_4_Cl, 2 mmol MgSO_4_, 0.1 mmol CaCl_2_, 0.1 µg thiamine, 100 mmol MOPS buffer pH 7.4, 3.7 µg (NH_3_)_6_Mo_7_O_24_·4 H_2_O, 25 µg H_3_BO_4_, 7.1 µg CoCl_2_, 2.4 µg CuSO_4_, 16 µg MnCl_2_, 2.8 µg ZnSO_4_, and 0.28 mg FeSO_4_. B32 cultures also contained 5 µg/mL tetracycline, 100 µg/mL carbenicillin and 34 µg/mL chloramphenicol and B86 cultures also contained 5 µg/mL tetracycline, 100 µg/mL carbenicillin, and 50 µg/mL kanamycin. Cultures were incubated overnight at 37°C and 250 rpm overnight and subcultured the following morning into the same medium containing the same antibiotics to an OD_600_ of ∼1.0 and allowed to grow to an OD_600_ of 3–5 at 37°C and 250 rpm. The cells were used to inoculate a 2 L bioreactor at 5% (v/v).

For shake flask production experiments 20 ml of M9 medium in 125 ml shake flasks was inoculated with frozen cells as described above. Following overnight growth at 30°C the culture was used to inoculate 140 ml of M9 medium containing 1 mM IPTG and 20 ml dodecane in a 500 ml flask at a 1∶100 dilution. Cell growth was monitored by OD_600_ while dodecane samples were removed at intervals for measurement of amorph-4,11-diene as described below.

### Fed-batch cultivation

The process used for production of amorpha-4,11-diene was based on the glucose process described by Korz et. al. [34]. One liter of batch medium composed of 4.2 g KH_2_PO_4_, 15.7 g K_2_HPO_4_·3H_2_O, 2 g (NH_4_)_2_SO_4_, 1.7 g citric acid, and 8.4 mg EDTA was prepared and sterilized in a 2 L Applikon Bioconsole ADI 1025 vessel at 121°C for 30 minutes. Post sterile additions for a batch medium were prepared as concentrated stocks, filter sterilized, and injected into the bioreactor through a septum on the head plate. They consisted of 15 g glucose, 1.2 g MgSO_4_·7H_2_O, 4.5 mg thiamine HCl, and 10 mL of batch trace metal solution containing 0.25 g/L CoCl_2_·6H_2_O, 1.5 g/L MnCl_2_·4H_2_O, 0.15 g/L CuCl_2_·2H_2_O, 0.3 g/L H_3_BO_4_, 0.25 g/L Na_2_MoO_4_·2H_2_O, 1.3 g/L Zn(CH_3_COO)_2_·2H_2_O, and 10 g/L Fe(III)citrate hydrate. Antibiotics (5 µg/mL tetracycline, 100 µg/mL carbenicillin, and 50 µg/mL kanamycin) were added to B86 processes for plasmid retention. For strain B32 plasmid stability was >95% without antibiotics in the production medium (data not shown). Cultivation was carried out at 30°C and the pH was adjusted at 7.0 using ammonium hydroxide. The airflow and the initial agitation rate were set at 1 v/v/m and 700 rpm, respectively. The dissolved oxygen tension was controlled at 40% of saturation using an agitation cascade and oxygen enrichment. Biospumex antifoam 200 K was used to control foam. Once the initial glucose (15 g/L) was exhausted and the dissolved oxygen spiked, an exponential feed with a 6 hour doubling time was initiated. The feed solution consisted of 650 g/L glucose, 12 g/L MgSO_4_·7H_2_O, 10.7 g/L (NH_4_)_2_SO_4_, 13 mg/L EDTA, and 10 mL/L of feed trace metal solution that was composed of 0.4 g/L CoCl_2·_6H_2_O, 2.35 g/L MnCl_2_·4H_2_O, 0.25 g/L CuCl_2_·2H_2_O, 0.5 g/L H_3_BO_4_, 0.4 g/L Na_2_MoO_4_·2H_2_O, 1.6 g/L Zn(CH_3_COO)_2_·2H_2_O, and 4 g/L Fe(III)citrate hydrate. (NH_4_)_2_SO_4_ was taken out from the feed for nitrogen restricted runs. The fermenter software (BioXpert) was programmed to calculate feed rates according to the following equation:
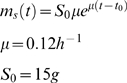
where S_0_ is initial glucose concentration, μ is specific growth rate, and t_0_ is time of glucose depletion from the batch medium. After reaching a maximum feed rate of 31 g glucose/h, the feed rate was reduced to 11.7 g glucose/h and held constant for the remainder of the run. The culture was induced with 1 mM IPTG at an OD_600_ of 30 and 10% (v/v) dodecane was added to the culture immediately after induction to capture amorpha-4,11-diene in the organic phase. The pH was maintained at 7 with the automatic addition of ammonium hydroxide and/or sodium hydroxide as described in the results. The fed-batch fermentation process was cultivated for 6–7 days.

### Ammonia, glucose, and acetate analysis

The ammonia, glucose, and acetate concentrations were measured off-line using Bioprofile 300 analyzer (Nova Biomedical) according to the manufacturer's instructions. Where the concentrations of the analytes were outside the range of the instrument, samples were diluted into Nova buffer which contained per liter 6 g HEPES acid, 0.64 g LiCl, and 0.46 g LiOH.

### Cell density measurements

OD_600_ was measured in a Genesys 10vis spectrophotometer at 600 nm. The relationship between dry cell weight and OD_600_ was measured in the following manner. Samples were taken throughout high density *E. coli* processes and OD_600_ was measured for each sample (the OD_600_ measurements ranged between 0.5 and 200). To measure dry cell weight, 1.5 mL of well mixed broth was centrifuged in weighed eppendorf tubes. The supernatants were removed from the samples and discarded. The cell pellets were washed once with water. The samples were centrifuged again and the water was removed. The washed cell pellets were dried in an oven at 80 C for at least three days. The samples were weighed, and the tube weight subtracted. The dry cell weight was calculated by dividing the sample weight (g) by the sample volume (0.0015 L), and graphing the relationship. OD_600_ of 1 was found to be equivalent to 0.4 g/L dry cell weight.

### Amorpha-4,11-diene quantitation

The production of amorpha-4,11-diene (AD) was monitored by gas chromatography/mass spectrometry (GC/MS), using trans-caryophyllene (TC; Sigma Chemical Company) as an internal standard. AD was extracted from the cell pellet by diluting 25 µL of well mixed whole cell broth with 975 µL of methanol, followed by mixing, and dilution of 10 µL of the methanol extract into 990 µL of ethyl acetate containing TC (10 ppm v/v). AD standards were prepared by diluting purified AD into the same ethyl acetate containing TC to concentrations between 0.63–10 mg/L. The standards and ethyl acetate-extracted samples were analyzed on an Agilent 6890N gas chromatograph equipped with an Agilent 5975 mass spectrometer (GC/MS) in single-ion monitoring (SIM) mode. The fragment ion, *m/z* 189, and the molecular ion, *m/z* 204, were used to monitor AD and TC. To expedite run times, the temperature program and column matrix were modified to achieve optimal resolution and the shortest overall runtime (5.43 min). A 1 µL sample was split 1∶20 and was separated using a HP-5MS column (Agilent Technologies, Inc.) with helium as the carrier gas. The temperature program for the analysis was follows: the column was initially held at 100°C for 0.75 min, followed by a temperature gradient of 65°C/min to a temperature of 170°C, where the gradient was decreased to 50°C/min until the temperature reached 300°C. The column was held at 300°C for 1 min to elute all remaining components. Under these conditions, TC and AD elute at 3.37 and 3.49 min respectively.
